# Unilateral versus bilateral thyroarytenoid Botulinum toxin injections in adductor spasmodic dysphonia: a prospective study

**DOI:** 10.1186/1746-160X-5-20

**Published:** 2009-10-24

**Authors:** Tahwinder Upile, Behrad Elmiyeh, Waseem Jerjes, Vyas Prasad, Panagiotis Kafas, Jesuloba Abiola, Bryan Youl, Ruth Epstein, Colin Hopper, Holger Sudhoff, John Rubin

**Affiliations:** 1 The Royal National Throat, Nose and Ear Hospital, London, UK; 2 UCLH Head & Neck Centre, London, UK; 3 Department of Surgery, University College London Medical School, London, UK; 4 Department of Oral Surgery and Radiology, School of Dentistry, Aristotle University, Greece; 5 Department of Medicine, University College London Medical School, London, UK; 6 Department of Otorhinolaryngology, Head and Neck Surgery, Klinikum Mitte, Bielefeld, Germany

## Abstract

**Objectives:**

In this preliminary prospective study, we compared unilateral and bilateral thyroarytenoid muscle injections of Botulinum toxin (Dysport) in 31 patients with adductor spasmodic dysphonia, who had undergone more than 5 consecutive Dysport injections (either unilateral or bilateral) and had completed 5 concomitant self-rated efficacy and complication scores questionnaires related to the previous injections. We also developed a Neurophysiological Scoring (NPS) system which has utility in the treatment administration.

**Method and materials:**

Data were gathered prospectively on voice improvement (self-rated 6 point scale), length of response and duration of complications (breathiness, cough, dysphagia and total voice loss). Injections were performed under electromyography (EMG) guidance. NPS scale was used to describe the EMG response. Dose and unilateral/bilateral injections were determined by clinical judgment based on previous response. Time intervals between injections were patient driven.

**Results:**

Low dose unilateral Dysport injection was associated with no significant difference in the patient's outcome in terms of duration of action, voice score (VS) and complication rate when compared to bilateral injections. Unilateral injections were not associated with any post treatment total voice loss unlike the bilateral injections.

**Conclusion:**

Unilateral low dose Dysport injections are recommended in the treatment of adductor spasmodic dysphonia.

## Introduction

Adductor spasmodic dysphonia (ADSD) is a focal dystonia of the laryngeal musculature, causing abrupt, intermittent and involuntary vocal folds spasms producing a strained and strangled speech pattern. It is idiopathic in nature and may reflect abnormalities in central motor processing [1].

The cardinal signs of ADSD are effortful vocal straining and harshness, quaver and voice arrest due to laryngospasm in the midst of non-effortful phonatory periods. It is described as "speaking whilst being strangled". Examination of the larynx may reveal true and false vocal folds hyper-adduction with laryngeal elevation and its attendant effects on speech. ADSD, a disabling disorder of voice, is characterised by involuntary disruption of phonation with functional, social and emotional consequences [1].

Botulinum toxin is the treatment of choice for ADSD and has been in use since the late 1980's [2-5]. It improves the patients' perception of dysphonia, mental health and their social function [6]. The American Academy of Otolaryngology-Head and Neck Surgery recognizes treatment with Botulinum toxin as the primary treatment for the ADSD (Policy statement: Botulinum Toxin; Reaffirmed March 1st, 1999).

Botulinum toxin inhibits the release of acetylcholine at the neuromuscular junction, causing a chemical denervation, thus resulting in muscle weakness or even paralysis in a reversible but long standing manner. The toxin has seven serotypes (A-G) [7,8] of which type A is commercially available and used as Botox^® ^and Dysport^® ^formulations.

Over the past two decades, in the absence of standardized guidelines, the dosing requirements for Botulinum toxin therapy for unilateral and or bilateral injections has varied significantly, both between patients and between injections in any one patient. Unilateral Botulinum toxin doses reported to vary between 2.5 mouse units (mu) [6], 4.0-4.5 mu [9,10], 5 mu [11], 15-16 mu [10,12,13], and 30 mu [14].

Bilateral doses reported to vary between 2.0 mu [10] and 2.5 mu [6,9,11,13] for each side. Both unilateral and bilateral thyroarytenoid muscle injections have been reported to be successful. To date, published literature has been inconclusive in comparing their effectiveness [2-5,10,14].

Commercially Botulinum toxin A is available as Botox^® ^and Dysport^®^. Botox^® ^is manufactured in the US by Allergan Pharmaceuticals (Irvine, California, USA) and Dysport^® ^is manufactured in the UK by Ipsen Products (Maidenhead, Berkshire, UK). Botox^® ^is available as 100 units/vial of frozen lyophilized toxin (stored at -5°C, $281.22). The toxin is shipped from the distributor in dry ice and is stored in a freezer at -5°C until reconstitution. Dysport^® ^is available as 500 units/vial (stored at 2-8°C, $308.13). Dysport^® ^is the formulation generally used in the UK hospitals.

A randomised controlled trial of Botox^® ^and Dysport^® ^suggested that Dysport^® ^tends to have higher efficacy, longer duration, and hence higher frequency of adverse effects [15]. The exact conversion factor for equivalence between the preparations is varied (site specific) and remains controversial. In clinical use in the larynx it is suggested that 1 Unit of Botox^® ^is approximately equal to 3 Units of Dysport^® ^[15].

In this preliminary study, we prospectively captured data and compared unilateral and bilateral thyroarytenoid muscle injections of Botulinum toxin in 31 patients with adductor spasmodic dysphonia, who had undergone more than 5 consecutive Dysport^® ^injections (either unilateral or bilateral) and had completed 5 concomitant self-rated efficacy and complication scores questionnaires related to the previous injections. We also attempted to address whether treatment should be administered unilaterally or bilaterally and also the dose of Dysport^® ^that produces the optimal clinical benefit. To improve communication between disciplines (in this case neurophysiology and ENT) we developed a Neurophysiological Scoring (NPS) system which, for us, had utility in the treatment administration. This, however, was not under research in this article. The Dysport infiltrations were done under EMG-guidance. This scale was used on a non-inferential basis to assess optimum needle placement within the muscle before administration of the injection.

## Materials and methods

This prospective study was carried out in the Royal National Throat, Ear and Nose Hospital, London, UK. Data were collected prospectively on a specific proforma from 1998 to 2006; 68 patients (42F/26M) with ADSD who had more than 5 consecutive Dysport^® ^injections were identified. However only 31 of those patients had either unilateral or bilateral thyroarytenoid muscle injections; the rest (37 patients) had a combination of both unilateral and bilateral injections (cross over). All diagnoses were made by a multi-spectral analysis (including laryngoscopy, voice analysis and speech therapist assessment) by the multidisciplinary team with due consideration to differential diagnoses.

An information sheet explaining the procedure in simple non-scientific terms was given to each of the patients. Each patient was asked to sign a consent form prior to treatment. The trial protocol was approved by the local committee of the ethics for human research.

Inclusion criteria were patients who have had only unilateral or bilateral Dysport^® ^injections for ADSD and continued on the same regimen for at least five consecutive treatments. Also, patients needed to be more than 18 years of age. Patients were excluded if they crossed over in treatment regimes (received unilateral and bilateral injections), had ADSD with vocal tremor or had previous laryngeal surgery or trauma. Pregnant women were not included in this study.

The diagnosis was made in a joint laryngology-neurology clinic. The patients were the major factor in influencing the choice of the course of their treatment regime. Since both the dosage and the side(s) of the injections were determined by clinical judgment and patient choice based on their previous injection clinical response and side we again point out that this was not a randomized controlled study. Clinical judgment mainly affected needle placement and dosage. The time intervals between injections were also patient driven since patients attended when their subjective voice quality deteriorated. The time interval between injections was determined by comparing dates of the previous and current Dysport^® ^injections. We reviewed 5 injection episodes with 4 time intervals and 5 pre-injection voice and neuro-physiological scores. Assessment was immediately before the next potential treatment episode.

In summary: the study includes patients who received five consecutive unilateral or bilateral injections. Once therapeutically stabilised outside of these five injections, no patient crossed-over treatment regimes. These five injections were not recorded from the first ever Botulinum toxin injection that each patient received. The initial dose and side per patient determined based upon previous responses and patient wishes.

Data were gathered on the response to treatment and the side effects to the previous injection (Table 1) on a standardised proforma developed by the authors. Each patient was asked to rate their best vocal quality following the previous injection on a six-point scale (Table 2).

**Table 1:** Complications of Botulinum toxin injection

**Side effects**
Total voice loss
Breathy voice "whispery"
Cough
Dysphagia

**Table 2:** Patient self-assessment of Voice Score

0	No improvement
1	Very slight improvement
2	Slight improvement
3	Moderate improvement
4	Marked improvement
5	Extreme/near normal

### The technique

Botulinum type A in the formulation of Dysport^® ^was used in all patients. Sequential dilutions with saline were performed until 2.5 mu were present in each 0.1 ml saline. There was no variation in the drug concentration used. All Dysport^® ^injections were performed using an insulated low profile fine bore 27-gauge mono-polar needle by or under the direct supervision of the laryngologist. No local anesthetic was used.

**Figure 1:**
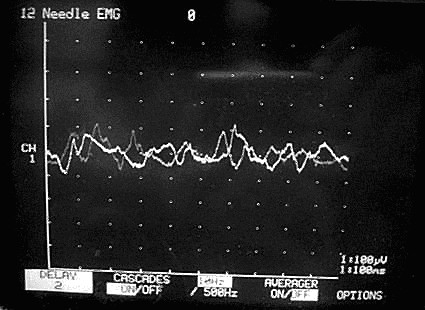
**Monitor showing NPS score of 2**.

**Figure 2:**
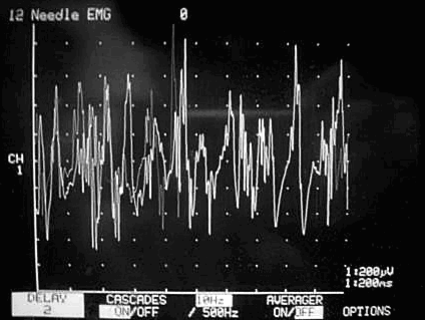
**Monitor showing NPS score of 9**.

Our technique is to insert the needle through the cricothyroid membrane and then angle it upwards and laterally to enter the thyroarytenoid muscle. The accuracy of the needle placement was confirmed by electromyographic (EMG) evidence of the characteristic waveforms during sustained phonation of a vowel sound (Figures 1 and 2).

In order to improve communication between the neurophysiologist and the injecting surgeon we have developed a 10-point subjective Neurophysiological Scoring (NPS) system to describe the amplitude and specificity of the EMG response (Table 3), where the NPS 0 to 5 (Figure 1) represents increase in distant motor activity and 5 to 10 (Figure 2) represents rise in the local muscle field motor activity on EMG. The NPS was recorded for each injection; all the NPS recording were carried out by the same team, in order to maintain consistency and a standardized interpretation of the EMG waveform.

**Table 3:** The neurophysiological score (NPS) and its electromyographic pattern

**NPS**	**Electromyographic pattern**
0	No motor activity
1	No local motor unit activity, few distant motor units
2	No local motor unit activity, moderate distant motor units
3	No local motor unit activity, abundant distant motor units
4	Occasional local activity, abundant distal motor units
5	Few low amplitude low interference pattern local field motor unit activity. moderate distal motor units
6	Moderate low amplitude low interference pattern local field motor unit activity, few distal motor units
7	Abundant low amplitude low interference pattern local field motor unit activity, no distal motor units
8	Half amplitude half interference pattern local field motor unit activity, no distal motor units
9	Near full amplitude near full interference pattern local field motor unit activity, no distal motor units
10	Full amplitude, full interference pattern local field motor unit activity, no distal motor units

### Statistical analysis

SPSS version 14 and Graph Pad Prism 4.0 statistical software packages were used. Student t, Chi Squared and ANOVA tests were used where appropriate with a significance level of p < 0.05.

We recognize a number of statistical assumptions that were made:-

• A sufficient washout period occurred between each patient consultation.

• There was no cumulative effect of treatment either in local drug accumulation, motor end plate loss, needle track scarring or fibrosis from previous haematoma formation.

## Results

**Figure 3:**
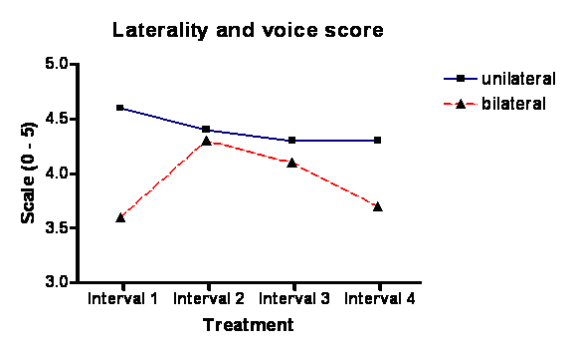
**Graph of voice score and laterality**. Showing a statistically significant trend for the unilateral injection group to have a better voice score over the interval between visits.

**Figure 4:**
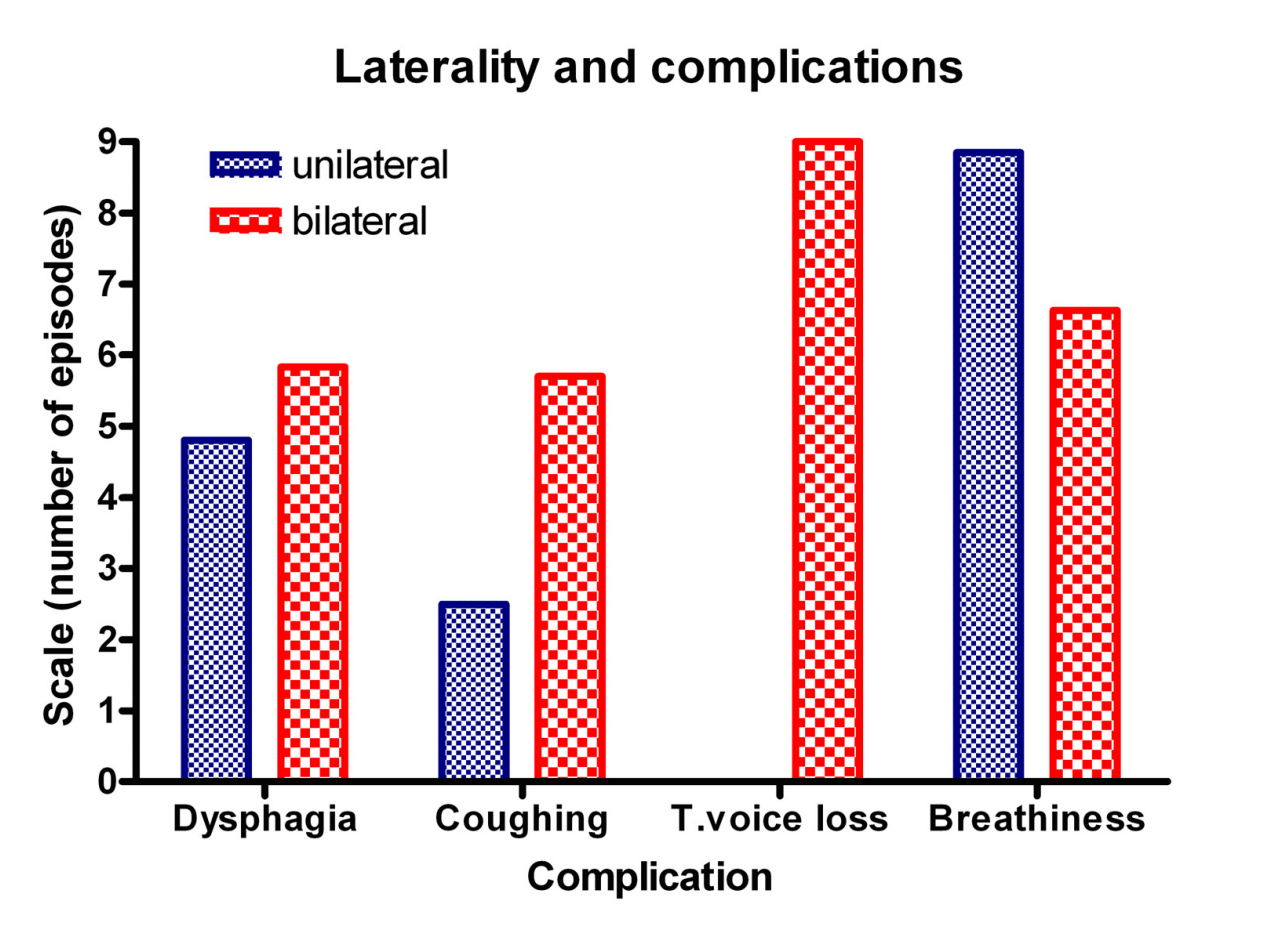
**Graph of episodes of complications and laterality of injections**. Showing that the unilateral injections group experience no total voice loss and are otherwise not statistically significantly different to the bilateral injection group.

**Figure 5:**
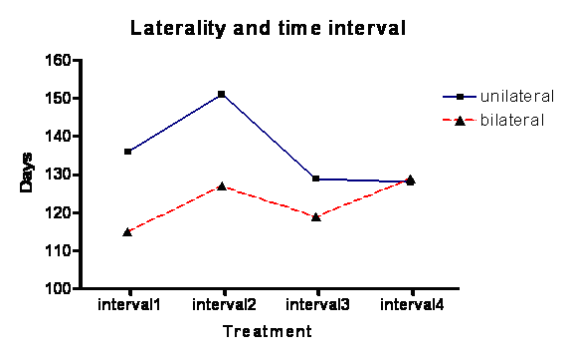
**Graph showing the average interval between presentations and the laterality of injections**. The unilateral injection group has a trend to a longer period between visits.

**Figure 6:**
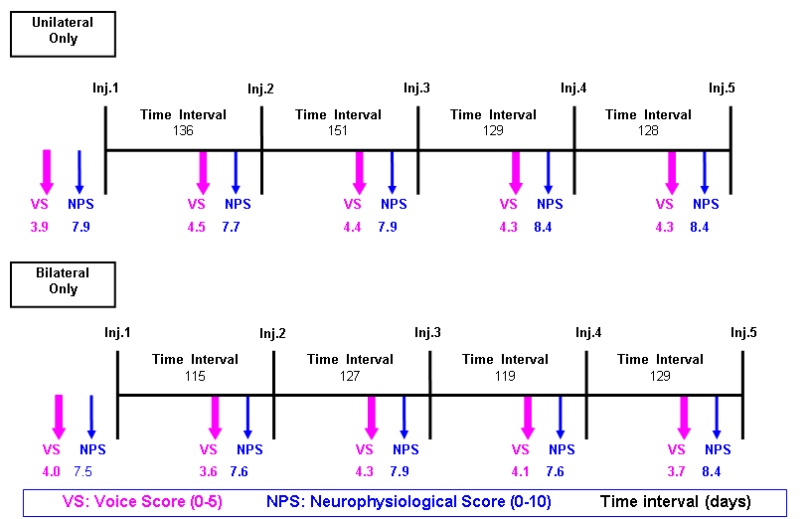
**Schematic summary showing average values for voice score (VS), neurophysiological score (NPS) and time intervals between attendance for injection in the unilateral and bilateral injection group**.

Thirty-one patients (16 females/15 males) with ADSD, who had undergone at least 5 consecutive Dysport^® ^injections were included in our study (Table 4); those patients had completed 5 concomitant self-rated efficacy and complication scores questionnaires (Figures 3, 4, 5, 6).

**Table 4:** Profile of treated cases (31 patients)

**Category**	**Unilateral group**	**Bilateral group**
**Gender**		
Male	6	9
Female	5	11
		
**Age (years)**		
Mean	55.65	58.52
Median	63.52	60.54
SD	15.45	16.03
		
**Injection episodes**	56	95
		
**Mean interval between injections (days)**	136.05 ± 56.2	122.68 ± 52.04
		
**Mean voice score**	4.24	3.93
		
**Complications**	26 episodes	59 episodes
Total voice loss	0	9 ± 5.73 (n = 6)
Breathy voice "whispery"	9.31 ± 7.76 (n = 16)	6.57 ± 4.88 (n = 35)
Cough	2.4 ± 0.89 (n = 5)	5.45 ± 6.23 (n = 11)
Dysphagia	4.8 ± 5.26 (n = 5)	6 ± 4.65 (n = 7)

There were total of 151 injection episodes, 56 unilateral and 95 bilateral. Eleven patients received unilateral treatment and 20 bilateral treatments. No patient had their first injection of Dysport^® ^within our study (Table 4).

The mean Dysport^® ^dose was 3.6 ± 0.02 units (~1.2 Botox^® ^units) for unilateral injection group compared to the mean total dose of 6.6 ± 0.02 units in bilateral injection group. The mean interval between injections for the unilateral group was 136 days as compared to 122 in the bilateral group. No patient expressed any deterioration in their voice. All the patients in the unilateral group and 90% of the patients in the bilateral group had a voice score of more than 2. The average voice score of 94% is consistent with other published results reported [1-4,14,16].

Our study revealed greater mean dysphagia duration and a shorter breathiness with the bilateral injections (Figure 4) but the difference in duration of these complications between unilateral and bilateral treatments were not statistically significant.

There was no significant statistical difference between unilateral and bilateral groups, which both had similar rates of complications. However, the unilateral group has a trend to lower complication rate as would be expected. Despite our assumptions the data has passed normality testing with an alpha score of less than "0.05". There was a significant difference in the mean voice score (VS) between unilateral and bilateral groups with the unilateral groups doing better (Student t test p < 0.05). This is also confirmed on one way ANOVA test. Even though there is a trend with unilateral injections having a longer duration of action this is not statistically significant. The total voice loss may be dose related since the dosing in bilateral patients was double the dose in unilateral patients (6.6 vs. 3.6), which could easily account for the 9 episodes of total voice loss in the bilateral group with none in the unilateral group.

## Discussion

In this study, our patients had either unilateral or bilateral thyroarytenoid muscle injections. As expected the 'real life' situation is reflected by the fact that we had a crossover group who had a combination of unilateral & bilateral injections; those patients were excluded from this study. Exclusion was considered necessary in order to compare pure injections groups. The patients in this group represent those who were not satisfied with their previous treatment. Hence our analysis group may reflect a satisfaction bias.

The unilateral injections compared to bilateral injections have less discomfort, less voice loss and reduced total drug used. However, historically, bilateral injections were used because the unilateral injections at that time were not satisfactory. There are disadvantages of giving any injection; these include discomfort and scarring of the injection tract. This may later have adverse effects on voice, which intuitively would be worse as the numbers of injections increases. Patients' subjective perception of treatment success was a significant indicator of outcome in this study. Patients' prime concern is less likely to be the instrumental measurements and more likely to be about their experience in daily life. It would have been desirable to also have an objective quantification of vocal changes. However, within the constraint of our study this was not feasible. Patients may have under or over reported their best voice score and complication duration due to recall bias. Using patient diaries can partially tackle this problem however we will then face the problem with patient compliance with diary completion as it drops over time [13]. As treatment was to a large extent patient driven and it is not feasible to blind them to treatment, patient reporting bias can not be totally eliminated.

The efficacy/benefit and complications profile were not significantly different. The unilateral injection involved only a single needle puncture and as would be expected from a single sided treatment is not associated with total voice loss. Furthermore it should be noted that the total volume of Dysport^® ^is increased in bilateral injections without a commensurate improvement in outcome. The equivalence of bilateral and unilateral injection outcomes may be explained in neurophysiological terms. Reduced afferent feedback may cause a compensatory reduction in efferent signaling to the contra-lateral larynx, however we cannot support this rationale hence essentially the findings remain unexplained. We feel that this would be the subject of further physiological experimentation.

We used a lower equivalent dose of Botox^® ^preparation (Dysport^®^) than in many published studies but with similar effectiveness [1-4,14,16]. It is possible that this is due to more exact needle placement guided by the electrophysiological waveform of the laryngeal adductors but we must assume that this is also carried out in other centres and the differences may simply be a statistical aberration.

Whereas some studies have shown a greater voice improvement and duration of response with the bilateral injections [9,11]; there have also been studies suggesting that unilateral thyroarytenoid muscle injections are more effective with a consistent treatment effect/side effect profile than the bilateral injections [10,16]. Low dose Botulinum toxin, especially for the unilateral treatment in our institution compared favorably to those reported by other centers. This supports the cumulative dose theory propagated by others [17,18] and can also decrease the likelihood of developing resistance to the medication. Bilateral Botox^® ^doses reported generally vary between 2 & 2.5 mu per side, whilst for the unilateral injections has been much higher (2.5 to 30 mu per site). The amount of Dysport^® ^we used was considerably below that of published data with mean equivalent dose of 1.2 mu of Botox ^® ^compared to the range of 4-30 mu in the literature for unilateral therapy.

A systematic review of 22 studies, involving Botulinum toxin for treatment of spasmodic dysphonia showed no significant difference in magnitude of effect between the unilateral and bilateral injections [19]. Liu et al. and Zwirner et al. have also shown that unilateral and bilateral injections did not differ in their efficacy or duration in relieving spasms [13,14]. There have also been studies that showed no significant difference in magnitude of effect between the unilateral and bilateral injections but in comparison much higher doses were used for the unilateral treatments [7,13,14].

The unilateral injection reduces the duration of the procedure and therefore the discomfort; it also makes the use of local anaesthesia which may interfere with EMG unnecessary. Knowing that the unilateral group had a high NPS, this may empower the patient and may also have a psychological effect, though this study did not specifically look at the psychological state of the patient.

Another advantage of the unilateral injections is the reduction in discomfort and cumulative needle injury to thyroarytenoid muscle, which results in scarring and fibrosis. Another plausible theory is the changes in the central pathophysiology and or possible effect of the toxin in the presynaptic neuron [7,20]. Unilateral low dosage injections of Dysport^® ^proved as successful as low dose bilateral injections in the treatment of ADSD in this limited patient sample. However, as treatment was to a large extent patient driven, there may be a patient reporting bias. This, however, does not invalidate this empirical study and is in line with other published series [2,6,9,10,13]. No improvement (VS = 0) was seen in 8.3% of bilateral injections compared to 2.2% in the unilateral. Patients who did not improve were more likely to be retreated with either higher dose or, if treated previously with unilateral dose, bilateral dose. Although not specifically investigated, these patients are likely to be those with more 'difficult' technical aspects to the injections. This undoubtedly led to the slightly higher incidence of no response in bilateral vs. unilateral injections. It also may have caused a slight bias towards better results in unilateral injections. Boutsen et al. suggested that the unilateral injection method is associated with a better side effect profile [21]. In the literature, bilateral injections were found to cause more dysphagia adverse effect [11-14] and breathiness [9,11].

The total mean dose Dysport^® ^injected in our study was 3.6 mu for the unilateral and 6.6 mu (3.3 mu each side) for the bilateral injections. Clinically this corresponds to 1.2 mu Botox^® ^for the unilateral and the total of 2.2 mu (1.1 mu each side) for the bilateral treatments [15,22].

Thus it appears that a relatively lower dose Botulinum toxin A administered for the unilateral injection episodes in our institution, compared to those reported by other centres to produce comparable results. Our results may support the cumulative dose theory propagated by others [18,23,24].

Several reported series have examined the difference in outcomes between unilateral and bilateral injections, both in terms of voice improvement and side effects [25]. Many show a longer duration of the treatment effect with the bilateral [9-11], compared to the unilateral injections but some show the opposite [10]. Bielamowicz et al. showed that the unilateral injection has more optimal and consistent treatment effect/side effect profile [10] and reduced the spasmodic muscle bursts in both the injected and non-injected muscles significantly. This is related closely to improvements in the speech symptoms [26].

In our study, we were surprised to find that so many of our patients improved with a very low dose especially on injections to only one side. Interestingly, many centers are now using smaller dosages of Botulinum toxin for injections than were first used. This has been postulated to be due to a cumulative effect of toxin over time, requiring smaller dosage for similar efficacy [24,27].

One advantage to unilateral injections is the reduction in cumulative needle injury to the thyroarytenoid muscle, which results to scarring and fibrosis and possibly transaction denervation. The effects of this therefore require further investigation. In an experimental model progressive muscle atrophy was noted [28].

Another intuitive advantage to low dosage is decreased likelihood of development of resistance. This may just be theoretical in importance as such low cumulative dosage is given to the larynx. In torticollis, where much higher dosages are required, it is a more significant issue. Greene et al. found about 10% of patients treated for torticollis developed resistance to Botulinum toxin type A [17]. Although to date this has not shown in cases of ADSD, it has led others to look at other types of Botulinum Toxin [18].

Anatomically, there is no muscle fibre communication between the thyroarytenoid muscles on one side with the opposite side [26]. Therefore it is unlikely that changes are result of diffusion of Botulinum toxin to the non-injected side. The results suggest that changes in the central pathophysiology may play a role in changes in speech symptoms following treatment [26]. There is evidence that in dystonia, Botulinum toxin transiently changes mapping of muscle representation areas in the motor cortex, and reorganizes inhibitory and excitatory intracortical pathways, probably through peripheral mechanisms [29]. Moreno-Lopez et al. indicated that intracellular retrograde axonal transport of Botulinum toxin in motor neurons may occur, suggesting a possible effect of the toxin on the pre-synaptic neuron. Interestingly the number of bursts in the non-injected side was reduced to a greater degree in patients receiving smaller dosages [26]. This correlates with the good response to treatment to low dose unilateral injection in our study.

Some studies including a review of head-to-head, randomized, controlled trials of Botox^® ^and Dysport^® ^in primary palmar hyperhidrosis suggest that Dysport^® ^tends to have higher efficacy, longer duration, but higher frequency of adverse effects [22,30]. Conversion factors between the preparations are varied and remain controversial.

The neurophysiological score (NPS), a 10-point subjective scoring system, was used to describe the amplitude and nature of the EMG response. This provided an immediate feedback to the injecting surgeon, reducing the time taken for each injection and hence patient discomfort. Thus we found the use of local anaesthesia unnecessary, especially as it may interfere with the EMG signals [31]. However, the use of the NPS does not explain why there was no statistical difference in the mean complication duration in the unilateral and bilateral injection groups.

The NPS can help treatment planning as a disappointing result from one injection, in a patient who previously responded well, may be due to poor needle placement(s) [32]. There are certainly some patients whose anatomy makes injection technically more challenging, and a poor result from the last injection combined with a relatively low neurophysiological score at that injection might lead one to suspect technical failure [33]. Although our scoring system is largely subjective, by working consistently with the same neurophysiologist a level of reproducibility can be reached which allows for meaningful longitudinal comparison. An inter-observer reliability test is being carried out as part of another study.

Our study has assumed that there was a sufficient washout period such that one injection methodology did not affect the following. However this is not necessary so, as the cumulative dose theory propagated [18,24]. Since patients made their appointment for repeat treatments when their subjective quality of voice deteriorated, we assumed that the time intervals between the injection episodes were the effective duration of the therapy. However patients may have prolonged their injection intervals due to personal circumstances.

The use of Dysport^® ^preparation of Botulinum toxin A in our study may have been a confounding factor in obtaining acceptable voice scores with lower clinically equivalent dose compared to Botox^® ^[22,23,34]. Several studies have shown a greater efficacy for Dysport^® ^injections than Botox^® ^injections however with increased complication duration. In this application, this may have been a confounding factor and more research into pharmaco-biology of different Botulinum toxin A preparations is warranted. Furthermore, the type of injection (unilateral vs. bilateral), dosing, and toxin type (Dysport vs. Botox) are not the only variables that may influence outcomes after injection. Volumes of reconstitution as well as concentration are also factors that may influence outcome as well and adds to the difficulty in comparing different research studies.

Other critiques of this study include the effect of the learning curve on the technique which may skew towards unilateral injections over the study period. The subjective nature of the decision of unilateral vs. bilateral was heavily influenced by patient choice. This, however, does reflect the current vogue of patient centered care and decision making and has been similarly reflected by Liu et al [13]. Further negative skew could be attributed by self selection of patients who required further treatment and hence attendance for injection therapy. Further, it may be inferred that patients may have preferred one injection episode to two; however, this inference is not without clinical significance. The use of established objective voice-related quality of life questionnaires (i.e. V-RQOL, VHI...etc.) would help.

## Conclusion

We recommend the use of unilateral injections for the treatment of ADSD. The advantages of unilateral injections are that there is no total voice loss so the patient can phonate and less drug dose is used. Treatment planning should be tailored to individuals keeping an acceptable balance between symptom relief and side effects. Obviously, further prospective studies are warranted, perhaps incorporating the use of voice related quality of life V-RQOL.

## Competing interests

The authors declare that they have no competing interests.

## Authors' contributions

TU, BE, WJ, VP, BY: contributed to conception and design, carried out the study and literature research, manuscript preparation and manuscript review. PK, JA, RE, CH, HS, JR: contributed to conception and design, contributed to the manuscript preparation and manuscript review. All authors have read and approved the final manuscript.
